# Annotation and characterization of lesions in breast tomosynthesis images

**DOI:** 10.1093/rpd/ncaf177

**Published:** 2026-03-13

**Authors:** Magnus Dustler, Akane Ohashi, Hanna Tomic, Kristin Johnson, Sophia Zackrisson, Anders Tingberg, Predrag R Bakic

**Affiliations:** Translational Medicine, Diagnostic Radiology, Lund University, 205 02 Malmö, Sweden; Translational Medicine, Medical Radiation Physics, Lund University, 205 02 Malmö, Sweden; Translational Medicine, Diagnostic Radiology, Lund University, 205 02 Malmö, Sweden; Department of Imaging and Physiology, Skåne University Hospital, 205 02 Malmö, Sweden; Translational Medicine, Diagnostic Radiology, Lund University, 205 02 Malmö, Sweden; Translational Medicine, Medical Radiation Physics, Lund University, 205 02 Malmö, Sweden; Translational Medicine, Diagnostic Radiology, Lund University, 205 02 Malmö, Sweden; Department of Imaging and Physiology, Skåne University Hospital, 205 02 Malmö, Sweden; Translational Medicine, Diagnostic Radiology, Lund University, 205 02 Malmö, Sweden; Department of Imaging and Physiology, Skåne University Hospital, 205 02 Malmö, Sweden; Translational Medicine, Medical Radiation Physics, Lund University, 205 02 Malmö, Sweden; Department of Hematology, Oncology and Radiation Physics, Skåne University Hospital, 205 02 Malmö, Sweden; Translational Medicine, Diagnostic Radiology, Lund University, 205 02 Malmö, Sweden; Translational Medicine, Medical Radiation Physics, Lund University, 205 02 Malmö, Sweden; Department of Radiology, University of Pennsylvania, 3400 Spruce Str., Philadelphia, PA 19104, USA

## Abstract

Rapid adoption of artificial intelligence methods in breast imaging research emphasizes the need for large, appropriately curated image databases for development and validation. For digital breast tomosynthesis (DBT), there are few public databases with only limited lesion annotation. Recently, we have developed Malmö Breast ImaginG (M-BIG), a large database of 104 791 women screened at Skåne University Hospital, Malmö. M-BIG also includes all images from the Malmö Breast Tomosynthesis Screening Trial, MBTST of 14 848 women, with 139 biopsy-confirmed cancers from DBT screening. To annotate lesions in M-BIG, we designed a semi-automated custom software tool for DBT, and corresponding digital mammography (DM) images. A reader manually draws an outline; or marks nodes around the lesion which are automatically connected by an edge-following algorithm. Our custom tool enables detailed annotation of DBT and DM lesions, as opposed to the rectangular regions present in other published material, allowing extensive evaluation of tumor segmentation, and analysis of size and shape descriptors.

## Introduction

Digital mammography (DM) has been the *de facto* standard for radiological breast imaging, both for diagnostic use and in breast cancer screening. Over the last decade, pseudo-3D digital breast tomosynthesis (DBT) is challenging the status quo, being almost universally used for clinical work-up. Many screening programs are transitioning into using DBT as a primary breast screening modality, citing better cancer detection and improved recall, such as the USA where as of 2025 close to 50% of all mammography units are DBT and over 92% of all screening centers offer DBT [1].

Rapid adoption of artificial intelligence (AI) methods in breast imaging has emphasized the need for large, appropriately curated databases of clinical images for both development and validation. Additional information besides the images determines for what purposes a database can be used. For some applications, it is enough to have the images without any additional information, but usually, at least diagnostic or structural information—of some level of detail—is preferable. Additionally, for any application aiming at detection, it is important that the data is annotated, i.e. that the location of the appropriate finding is marked and possibly that the outlines are indicated. Having access to lesion outlines allows the extraction of quantitative image features associated specifically with the lesion. This is important for both traditional radiomics and AI, as well as for hybrid approaches.

A relatively large number of high-quality databases are available for DM. For the more recently adopted DBT however there is a lack of representative and well-annotated databases, due to its limited use in screening.

A search for publicly available databases with annotated DBT images turned up two candidates: the Duke DBT Database [2, 3] from the USA and the OPTIMAM Mammography Image Database from the UK [4]. The Duke database includes a screening population of 5060 women, where all biopsy-confirmed malignant and benign findings—89 cancers and 112 benign lesions—are annotated in one DBT slice with a rectangular bounding box. OPTIMAM is an enriched data set with 1568 cancers and 130 benign lesions out of 5790 examinations. Like in the Duke set, all cancers and benign findings are marked with a rectangular bounding box.

The paper describes the methodological steps necessary for the annotation of breast lesions and their influence on the development of a user-friendly annotation tool that can be used to mark and record lesion outlines and locations as well as relevant radiological variables in mixed DBT and DM datasets. The tool allows for easy modification and review of the results and progress.

## Methods

### Dataset

The Malmö Breast ImaginG database, M-BIG, is a large database containing mammographic images of 104 791 women imaged at Skåne University Hospital, Malmö, between 2004 and 2020. It includes all mammographic images of these women, in total about 500 000 examinations, with a mix of screening and diagnostic images [5]. M-BIG also includes all images from the Malmö Breast Tomosynthesis Screening Trial. MBTST ran from 2010 to 2015 and included 14 848 women who underwent paired DM and DBT imaging that were read in two different screening arms [6]. This provides a unique dataset of DBT-screening data. Each case has at least 2 years of follow-up, providing detailed data on interval cancers. In total, 158 biopsy-proven breast cancer cases, including interval cancer, are included in the dataset. In normal screening mammography, two projection views of each breast are normally acquired—CC and MLO (medio-lateral oblique).

The database images are stored in Digital Imaging and Communications in Medicine (DICOM)-format in a secure server environment.

### Software tool design

To annotate lesions, we designed a custom software tool focused on semi-automated identification of lesion outlines in DBT and corresponding DM images. The annotation tool was developed in Matlab (Mathworks, Natick, USA), using the built-in GUI (graphical user interface) design package and was packaged and deployed both as a self-contained executable and as an app launchable from inside Matlab. It was tested to run in a Windows 11 environment. Input images should be stored in a single directory, with each subdirectory containing all DICOM-images linked to a specific case. The tool automatically detects the laterality and view of each image.

**Figure 1:**
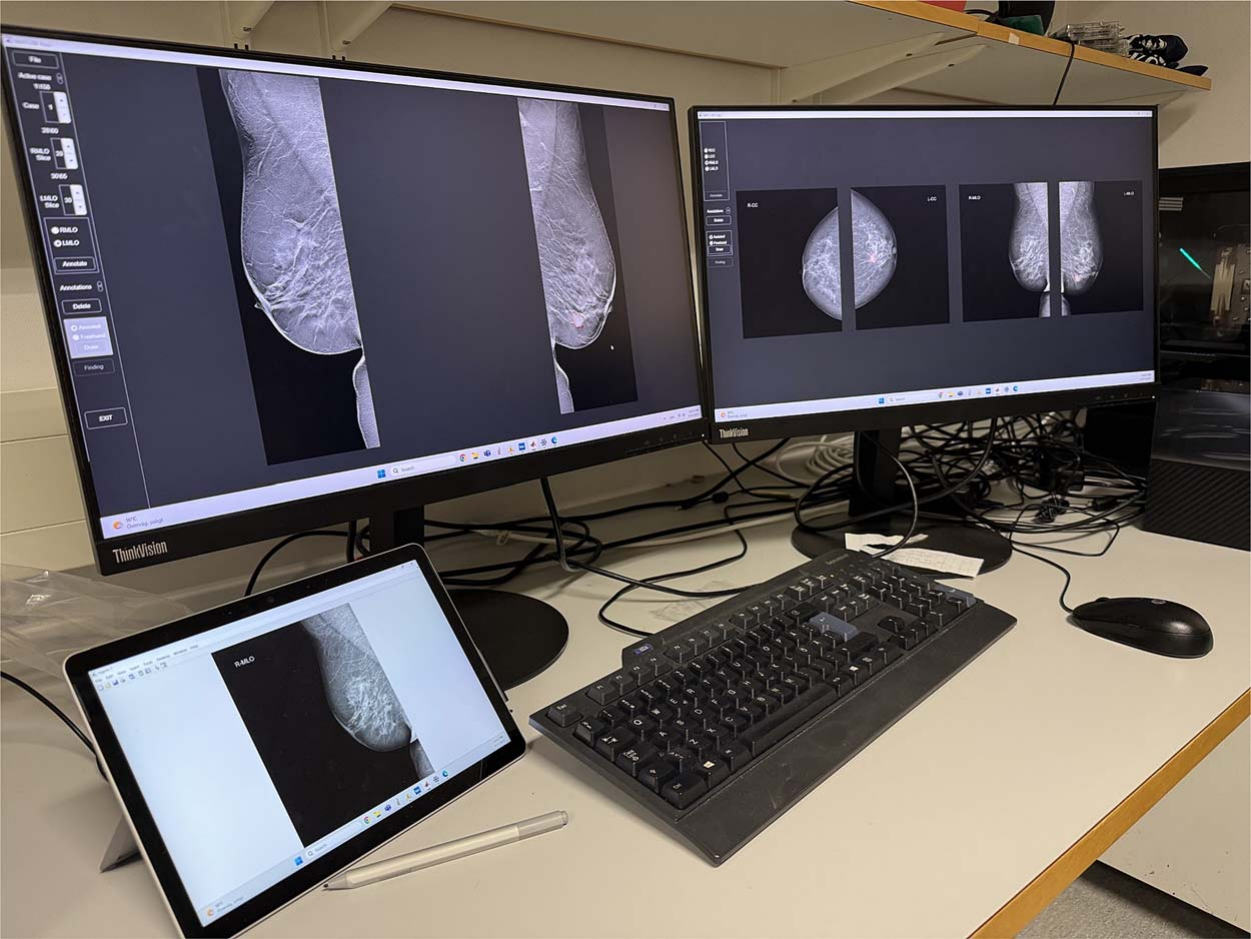
The Matlab-based GUI deployed and installed on a workstation: A case is open, with DBT MLO images displayed on the left screen and corresponding DM images of the same case in cranio-caudal and MLO projection of the right, while the small screen is a linked tablet where an annotation window of the select case has automatically opened so that the outlines can be marked with the stylus.

The software tool was designed specifically for the paired format of the MBTST data, using a three-screen setup, though this is easily and flexibly adaptable to other datasets with other requirements for hanging protocols. Two high-definition, high-resolution monitors are used to display mammographic images and taskbars, while a linked touch-screen tablet is used to draw annotations. DBT images were displayed on one screen and DM images on the other. The Windows Surface Tablet was tethered through a wireless connection to function as a third screen, allowing the use of a stylus for precise drawing on its touchscreen. See Fig. 1 for an illustration of the setup with a loaded case.

A reader has the option of either manually drawing a lesion outline (based on the Matlab *freedraw()* function) or, alternatively, to place nodes or waypoints around the outline of the lesion which are then input into an automatic edge following algorithm [7] which connects waypoints around the lesion outline (based on the Matlab *drawassisted()* function which uses the intelligent scissors method). In either case, the lesion outlines are displayed along with a set of waypoints which can then be manually adjusted, added to, or removed. Both drawing options support either a standard mouse or a stylus that can be used alongside the touchscreen.

Annotation data is automatically and dynamically saved as variables in a Matlab file (.mat), which is in turn stored in the relevant subdirectory. The individual files include lesion morphological information (see below), information about which slice the lesion is on (for DBT) and a vector of outline coordinates. By design, original DICOM-images are not altered in any way, and their metadata is kept intact. The information in the annotation .mat-file can be converted to and exported as binary, text or csv files.

### Setup and use

**Figure 2:**
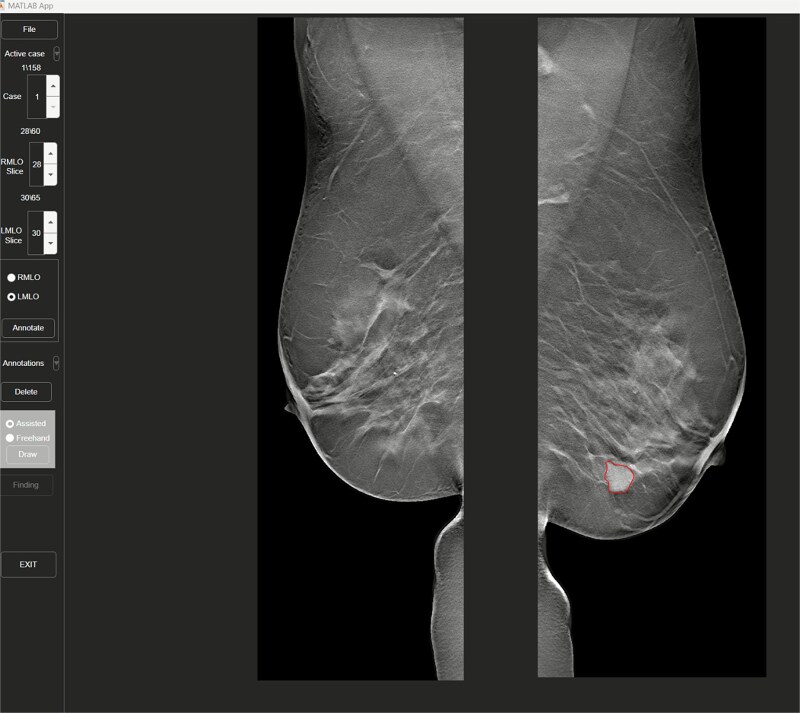
A cropped view of the reading window of the annotation tool, showing only the DBT screen. Note the annotated lesion in red.

**Figure 3:**
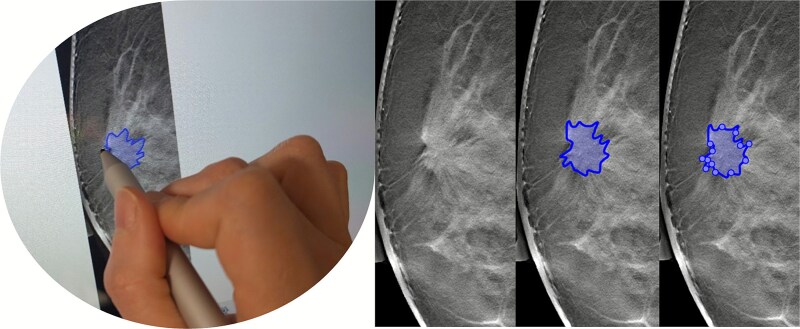
An illustration of the annotation tool in use, showing the stylus used to delineate a lesion, and the automatically calculated waypoints that can be used to adjust the outline.

Figure 2 shows an illustration of the GUI. It provides the following options for the reader:

- **File**: Through this menu the user browses available directories and selects a folder of cases. In this example, the folder contains 158 cases (the number of breast cancer cases from MBTST). The current case number is displayed just below the File button.
- **Case**: These arrow keys are used to switch between cases. A case can also be selected through the *active case* drop-down menu just above it. Selecting a new case leads to it being loaded and displayed on both screens in DBT and DM.
- **Slice**: For the DBT view, this number displays the current slice for the respective 3D stack of images (RMLO and LMLO). Pressing the buttons will automatically go to the next and previous slice, as will using the arrow keys or the mouse wheel (for the selected image).
- **Annotate**: Pressing this button will bring up an annotation window on the touch screen (Fig. 3), allowing either **Assisted** or **Freehand** annotation, as described above. After annotation is done and **Finding** is pressed, a further pop-up window is displayed allowing the selection of lesion morphological information. Each lesion type (mass, microcalcification, architectural distortion, and asymmetry) is visually indicated by a distinct outline color.
- **Annotation**: This dropdown menu displays a list of previously saved annotations on the select case, and selecting one of them will automatically jump to the correct slice. Selecting an active annotation also activates the **Delete**-button which allows individual annotations to be removed.

### Breast lesion annotation and categorization

In a workflow, the reader identifies lesions directly on the images, if possible on both DBT and DM of the same case. For non-calcified lesions, the recognizable boundary area was identified and annotated; for calcified lesions, the area was more roughly outlined, rather than marking individual calcifications. If a lesion was unclear, difficult to identify, or not visible, the PACS system was used to verify the location of the biopsied lesion.

To investigate the relationship between the information annotated for the lesions and the morphological diagnosis by radiologists, morphological evaluation was performed and recorded using the Breast Imaging Reporting and Data System lexicon [8] of mammography, which is used for clinical imaging diagnosis of breast lesions. The descriptors include mass (shape, margin, and density), calcification (morphology and distribution), architectural distortion, and asymmetry.

## Results and discussion

### Annotation results

Our evaluation with cancer DBT sets from MBTST suggests the tool is found by radiologists to be easy to use and that the annotation style can be flexibly adapted to the most appropriate case. One radiologist has annotated >100 cancer cases in DBT and DM. Annotation is ongoing for all biopsy-confirmed cancers and benign tumors. Except in some rare cases, interval cancers were not annotated as they could not be clearly identified. Certain lesions were visible only on DBT and not on DM. In general, the annotation itself using the tool was quick (~1 min per case), with most of the time spent locating the correct lesion through cross-referencing PACS records.

The contrast between the lesion and background breast tissue also affects identifying and annotating lesions, which can be particularly difficult in the case of dense breast tissue. Having both modalities, DBT and DM, available in the same workflow makes it easier to detect lesions, improving consistency. Due to morphological features like architectural distortion, asymmetry, or obscured/indistinct margins, clearly defining lesion borders can be challenging. A freehand drawing approach may suit these lesion types to encompass the findings. In contrast, well-defined lesions may be better annotated using an assisted method that traces their outlines. In addition, using this method, calcified areas were roughly outlined; however, future work should consider extending the annotation methodology to allow marking individual calcifications.

### Tumor size measurements

As a simple validation experiment, we calculated tumor sizes based on the annotated lesion Regions of interest (ROIs) and compared them to pathological tumor sizes from MBTST clinical data. The tumor size was defined by calculating the Feret diameter of the annotation, i.e. the largest extent of the ROI. On average, the calculated lesion sizes from both DBT and DM annotations were larger than the recorded pathologic sizes, by an average of 2.0 ± 10.6 mm and 1.2 ± 9.9 mm respectively. The differing size may be an indication both of the different methods of measurement (assumed largest extent vs actual largest extent of the annotation) and of the different sources of size data (pathological slices vs radiography). Also, the tool can be used to estimate a lesion cross-section area by use of the ROI. The area measurement arguably provides a more accurate measure of lesion size than just the largest diameter. Measured area from annotation is on average smaller than that assumed from the recorded pathological size, if assuming a circular lesion approximation. The mean difference in size is 121 ± 343 mm^2^ for DBT and 114 ± 319 mm^2^ for DM, respectively. This large variation illustrates the importance of having access to more than one dimensional measurements in order to achieve a representative value for actual tumor size.

### Integration with other software—Pyradiomics

We have successfully exported the lesions outlines to an open source radiomics package, WEKA [9], creating ROIs that can be used for calculating standardized radiomics features, and for other related applications such as machine learning.

## Conclusion

Our custom tool enables the annotation of a detailed lesion outline of DBT and DM lesions, as opposed to the rectangular ROIs present in other published material. The presented tool enables efficient annotation of detailed lesion outline, a unique feature of M-BIG, which allows extensive evaluation of tumor segmentation, and analysis of lesion size and shape descriptors.

The tool in its current build is available as a free download via Mathworks file exchange https://se.mathworks.com/matlabcentral/fileexchange/182784-mammo-annotation-app. It is provided as-is, will be continuously updated and may not exactly correspond to the version described in this paper.
